# Natural History Studies in NCL and Their Expanding Role in Drug Development: Experiences From CLN2 Disease and Relevance for Clinical Trials

**DOI:** 10.3389/fneur.2022.785841

**Published:** 2022-02-08

**Authors:** Miriam Nickel, Angela Schulz

**Affiliations:** Department of Pediatrics, University Medical Center Hamburg-Eppendorf, Hamburg, Germany

**Keywords:** CLN2 disease, NCL, batten disease, natural history studies, rare disease (RD), drug development, neuronal ceroid lipofuscinosis, childhood dementia

## Abstract

Conducting clinical trials in rare diseases is challenging. In trials that aim to use natural history control cohorts for evaluation of efficacy, lack of data on natural history of disease prolongs development of future therapies significantly. Therefore, collection of valid natural history data in clinical settings is needed to advance drug development. These data need to fulfill requirements on type of collection, quantifiable measures on the course of disease, verification and monitoring as well as compliance to strict data protection and sharing policies. Disease registries can be a source for patient data. Late-infantile CLN2 disease is characterized by rapid psychomotor decline and epilepsy. Natural-history data of 140 genotype-confirmed CLN2 patients from two independent, international cohorts were analyzed in a natural history study. Both datasets included quantitative ratings with disease-specific clinical scores. Among 41 patients for whom longitudinal assessments spanning an extended disease course were available within the DEM-CHILD DB (an international NCL disease patient database, NCT04613089), a rapid loss of motor and language abilities was documented in quantitative detail. Data showed that the course of disease in late-infantile CLN2 disease is highly predictable with regard to the loss of language and motor function and that the results were homogeneous across multiple and international sites. These data were accepted by EMA and FDA as valid natural-history controls for the evaluation of efficacy in experimental therapies for CLN2 disease and led to an expedited approval of intracerebroventricular enzyme replacement therapy with cerliponase alpha in May 2017.

## Introduction

Degenerative brain diseases of childhood are a group of rare, heterogeneous diseases including neuronal ceroid lipofuscinoses (NCLs), some forms of mucopolysaccharidoses, leukodystrophies and other rare diseases of inborn errors of metabolism. The majority are relentlessly progressive, incurable and lead to early death. Because they involve loss of developmental skills and intellectual disability, many of them meet the criteria of “childhood dementia” (1, 2). Collection of valid natural history data in these diseases is challenging due to small patient numbers, phenotype variability, missing objective clinical outcome measures and limited disease specific clinical rating scales. Lack of these data prolongs development of future therapies significantly. Natural history of disease data (precisely quantifying progression of disease symptoms and their functional relevance) are therefore needed to advance therapy development.

### Overview of NCL Diseases

Neuronal ceroid lipofuscinoses are lysosomal storage disorders characterized by abnormal accumulation of autofluorescent material in lysosomes of the cells (3) and mainly affect retina and gray matter of the cerebral cortex (4). NCL diseases are monogenic diseases and – with one exception – inherited in an autosomal recessive mode. To date, genetic variants in 13 different NCL genes have been described (https://www.ucl.ac.uk/ncl-disease/mutation-and-patient-database). These genes code for lysosomal enzymes, membrane proteins located in different organelles, or other proteins (5). Exactly how deficiencies in these different proteins lead to neurodegeneration and accumulation of lysosomal storage material has not yet been clarified for any of the known NCL diseases. Classification of NCL diseases is currently based on the defective genes and age at first manifestation of symptoms (congenital, infantile, late infantile, juvenile or adult) (5–7). NCLs share common clinical features, in most cases a combination of epilepsy, psychomotor regression with cognitive decline and vision loss. Before onset of symptoms, patients show a largely normal psychomotor development. Most NCL gene defects are assigned a clearly recognizable “classic” or “typical” disease phenotype, which is suggestive of a complete loss of function of the affected protein. Apart from these, a growing number of patients are being identified with “atypical” phenotypes, caused by so-called “mild” genetic variants, that do not lead to a complete loss of function of the corresponding protein.

NCL diseases are diagnosed on the basis of clinical findings, laboratory tests for disease-specific enzymes, cell-morphology studies and genetic testing (4), aiming to identify the pathogenic genetic variants in both alleles of the NCL gene. The increasing use of Next Generation Sequencing Panels and exome sequencing as tools for the diagnosis of rare diseases may lead to the diagnosis of NCL in patients not previously suspected to have the disease. Due to these advancements in genetic testing, the number of atypical, often milder phenotypes described is rapidly increasing especially in NCL forms where a lysosomal enzyme is affected such as CLN2 disease (8–10). Electron-microscopic demonstration of lysosomal storage material in tissues or blood cells can be helpful for detecting an NCL disease. In juvenile CLN3 disease, light microscopy of a blood smear shows characteristic vacuolated lymphocytes. Enzyme testing is a quick and inexpensive way to screen for CLN1 or CLN2 disease. The activity of lysosomal enzymes can be measured in a dry blood spot sample (11). Lack of activity of the enzyme palmitoyl peptidase 1 (PPT1) confirms the diagnosis of CLN1 disease, lack of activity of tripeptidyl peptidase 1 (TPP1) that of CLN2 disease.

### Late-Infantile CLN2 Disease

CLN2 disease is caused by genetic variants in the *CLN2* gene that encode for a lysosomal serine protease, tripeptidyl peptidase 1 (TPP1) (12). Loss of TPP1 activity leads to accumulation of ceroid lipofuscin (13, 14). Although there is considerable allelic heterogeneity, two *CLN2* genetic variants are common to more than half of known CLN2 cases in the Western Northern Hemisphere: c.509-1G>A (splicing error) and c.622C>T (non-sense mutation leading to an early stop codon) (5). There is a high genotype-phenotype correlation regarding these variants, leading to a predominantly late-infantile phenotype. Many rarer *CLN2* genetic variants have been reported whose influence on phenotype and residual TPP1 activity is not well understood; some genetic variants are thought to result in the delayed onset or prolonged course of the disease (15). Atypical CLN2 phenotypes are reported more frequently, with increased ability for genetic testing from different regions in the world (8, 10, 15–22). In Latin America 50% of cases show protracted course of disease with later onset of symptoms and slightly different order of symptom onset (9).

Late-infantile CLN2 disease (OMIM# 204500) globally has been the most common phenotype in CLN2 disease. It is characterized by a severe and rapidly progressive neurodegenerative syndrome with onset most common in children aged 2–4 years (23). Epileptic seizures, loss of language, motor function and cognition, blindness and premature death (14, 24) quickly follow the onset of first symptoms. Photoparoxysmal response (PPR) on intermittent photic stimulation at low stimulation frequencies of 1-3 Hz can be an early hallmark (25). CLN2 disease occurs worldwide, but data on incidence and prevalence are scarce (26–28). Because the first symptoms (language delay, epileptic seizures, delay of psychomotor development and ataxia) are non-specific to CLN2 and are sometimes found in children with epilepsy as side effects of anti-seizure medications (ASMs), they are commonly misinterpreted, and diagnosis is often delayed. Vision loss, a characteristic clinical sign for NCL, occurs only in the late stages of late-infantile CLN2 disease and therefore is not a clinical hallmark for early diagnosis. The burden of CLN2 disease is high, not only emotionally for affected families but also for society at large. A missed diagnosis prevents parents from receiving genetic counseling for future planned pregnancies and may lead to their having subsequent children who are also affected.

## Natural History Data in Clinical Trials for Rare Diseases

### Regulatory Agency Recommendations on Natural History Data Used in Clinical Trials

Since the number of rare diseases is rising, with advanced diagnostic methods and genetic testing available, development of new drugs for the unmet medical need of millions of patients is needed. Natural history data can serve as control data in clinical trials and therefore shorten time in drug development. Therefore, regulatory agencies, such as FDA (29) and EMA (30), have released guidelines to ensure high quality of natural history data for drug development in rare diseases.

Key points of these guidelines are:

- DATA SUITABILITY: data collection, data storage, data extraction and quality control processes need to fulfill the basic rules of GCP (good clinical practice) guidelines for clinical trials. Any source data need to be dated and signed and digital records have to be traceable with audit trails. Most importantly, informed consent processes for participation in a scientific data collection and international data sharing need to be in place, considering local ethical requirements as well as privacy issues and governance-related issues.
- PATIENT POPULATION AND BIOMARKERS: genotypic and/or phenotypic heterogeneity can affect the characterization, progression, and physiological changes of the disease. Therefore, such information is important for the development of clinical biomarkers for diagnosis, prognosis of disease progression, and prediction of treatment response. In addition, it might guide patient and dose selection in clinical trials. Disease specific centers of excellence should guide these data.
- CLINICAL OUTCOME ASSESSMENTS: can assess both safety and efficacy and may include observer-reported, patient-reported, caregiver-reported, and performance outcome measures. Natural history studies can combine information from patient medical records and other existing sources of disease-specific information. Longitudinal retrospective and prospective (longitudinal) studies can help fill critical gaps in knowledge of disease progression and set a course for future analysis. In these studies data are collected over time, making them more suitable for use as an external control group. Cross-sectional studies collect patient data at a specific time point offering a snapshot of disease and can be used to support existing data.

EMA guidelines specifically focus on data collected in disease registries, which is defined “as an organized system that collects data and information on a group of people defined by a particular disease or condition, and that serves a pre-determined scientific, clinical and/or public health (policy) purpose”. In comparison, FDA guidelines are kept more general in use of natural history study data provided. Both guidelines share similar keypoints though regarding quality and use of data and encourage the use of clinically collected natural history data in rare diseases for use in drug development.

### Methods Established in Natural History Data Collection for NCLs

#### Disease Specific Center of Excellence

For over 20 years, the Hamburg Specialty Center for NCL and related childhood dementias has been established and is treating around 170 patients annually with all types of NCL diseases. The DEM-CHILD database, an NCL disease registry, was founded in Hamburg as part of the EU-funded FP7 project DEM-CHILD (“A Treatment-Oriented Research Project of NCL Disorders as a Major Cause of **Dem**entia in **Child**hood”, GAN°281234, www.dem-child.eu). The DEM-CHILD project focused on the main cause for childhood dementia in Europe, the neuronal ceroid lipofuscinoses (NCLs). In order to advance the development of treatment options for NCL diseases, the DEM-CHILD project combined the expertise of (i) recognized European research teams, both basic scientists and clinicians, (ii) high-technology SMEs, (iii) experts in medical ethics, and (iv) NCL patients and family associations. The project implemented a novel network including the most prominent NCL researchers, both basic scientists and clinicians, in Europe, collaborating with Indian experts, to collect the world largest, clinically and genetically best characterized set of NCL patients. One work package was (i) to establish an NCL mutation and NCL patient registry of long-lasting function to describe accurately and in detail the clinical course and clinical spectrum, as well as genotype-phenotype variability in different forms of NCL; and (ii) to establish a tool for the evaluation of experimental therapy studies in the NCLs.

#### The International DEM-CHILD Database

Only patients with a genetically verified diagnosis of an NCL disease can be included in the data collection after signing an informed consent for participation.

Following the successful application for an international natural-history study for all NCL diseases (www.clinicaltrials.gov, NCT04613089), the DEM-CHILD database is nowadays able to collect data internationally. For rare diseases, such global cooperation is desperately needed for building robust datasets for future therapeutic trials. Different types of data are collected within the DEM-CHILD database, separated into “static” and “dynamic” datasets. Static data do not change over time (e.g., genetic diagnosis, age at first symptoms and timepoints of reached milestones in psychomotor development as well as their loss) whereas dynamic data are related to an exam date and might change over time with progression of disease (Table 1). Examples for dynamic data are clinical scoring data on disease progression (e.g., disease specific clinical rating scales (Table 2) and all examinations conducted at a defined timepoint. The static data sets represent the background for the analysis of the dynamic data. They allow the interpretation of different disease progression rates based on the genetic diagnosis and thereby lead to a better understanding of the genotype-phenotype correlation. In addition, detailed analysis of the retrospective static data has led to the identification of early symptoms supporting early diagnosis.

**Table 1:** Overview of types of data (static/retrospective, dynamic/prospective).

**Static data (retrospective)**	**Dynamic data (mainly prospective)**
• Demographics • Genetic Diagnosis • Medical history (age at diagnosis, onset of symptoms, type of first symptoms) • Age at achievement of milestones of psychomotor development	• Disease specific rating scales (see Table 2) • MRI/OCT/EEG/other diagnostic evaluations • Clinical examinations (standardized neurodevelopmental exams, etc.) • QoL Questionnaires

**Table 2:** Overview of disease specific rating scales for NCLs.

			**Type of analysis possible**			
**Clinical rating scale (CRS)**	**NCL phenotype**	**Clinical categories**	**Retrospective**	**Prospective**	**No of patients in original publication**	**Reference**
Hamburg INCL score	infantile	Mobility Fine motor function Expressive language (add on categories: communication/interaction, sleep, agitation/irritability, seizures, feeding, visual attention)	✓	✓	14	(31)
Hamburg LINCL score	late-infantile	Motor Language Seizures Vision	✓	✓	22	(32)
Hamburg JNCL score	juvenile	Motor Language Epilepsy Vision Intellect	✓	✓	17	(33)
Weill-Cornell (WCMC)	late-infantile	Motor Language Gait Feeding	*X*	✓	18	(34)
CLN2 clinical rating scale ML (CLN2-CRS ML)	late-infantile	Motor Language	*X*	✓	24	(35)
Expanded CLN2 clinical rating scale (CLN2-CRS MX-LX)	late-infantile	Motor Language	*X*	✓	30	(36)
Unified batten disease rating scale (UBDRS)	juvenile	Physical Seizures Behavior Capability Clinical global impression	*X*	✓	31, 82	(37, 38)
Opthalmologic: Weill-Cornell LINCL opthalmic scale (WCBS)	late-infantile	Color/fundus OCT Fluorescein/indocyanine green angiogram (FA/ICGA)	✓^*^	✓	25	(39)
Opthalmologic: Hamburg CLN3 opthalmic rating scale	juvenile	Visual acuity/BCVA Fundus score OCT	✓^*^	✓	21	(40)
Pain: battens observational pain scale (BOPS)	not specified	Pain	*X*	✓	35	(41)

* *If images retrospectively have been collected for OCT, FA and ICGA*.

### Clinical Rating Scales in NCL Diseases

The development of new therapies requires an exact understanding of the clinical course of the disease needed to treat, and its variability across a wide range of patients. Limited quantifiable data make statistical analysis and extrapolation difficult. To date, only a few reports have provided a quantitative description of the clinical course of disease in NCLs. Disease specific clinical rating scales have been developed for infantile, late-infantile and juvenile phenotypes. Rating severity and progression of key symptoms of disease, these can be applied prospectively, retrospectively or combined, depending on the specific scale (Table 2). In addition, novel ophthalmologic scales also focus on rating progression of vision loss and changes in retinal function and histology as presented in Table 2 below.

### Role of Natural History Data in Drug Development for CLN2 Disease

Until recently, only palliative treatment had been available for CLN2 patients.

Therefore, the aim of a collaborative natural history data collection for CLN2 disease was to create a precise dataset on disease progression which should ultimately serve as control data for experimental therapy studies.

In an international natural history collection of 140 patients with late-infantile CLN2 disease (42), 41 patients from the DEM-CHILD DB from Hamburg could provide a longitudinal quantitative core data set, with clinical ratings throughout the long course of the disease. Patient cohorts from Italy (Verona) and the US (Weill Cornell Medical College) provided predominantly cross-sectional or short-time longitudinal data sets in addition. Acquired in a multi-site international setting, the findings demonstrated that the rate of disease progression was homogeneous for all data sets and that, despite subjects being located in different countries and being independently rated, the results were highly reproducible across the cohorts.

These CLN2 natural history data have successfully been used as natural-history controls in a clinical trial testing safety and efficacy of intraventricular enzyme replacement therapy with cerliponase alfa (43). Comparison of data from treated patients with these natural history data showed that this treatment was able to slow down disease progression. Subsequently, these data were monitored and approved as part of the market approval process of cerliponase alfa by EMA, FDA and PMDA.

## Discussion

Valid longitudinal and internationally collected natural history data, that can quantify disease progression in rare diseases, are urgently needed for evaluating the efficacy of current and future therapies and to shorten time to approval of new therapeutic options. Collection of these data face multiple challenges when used as comparative cohorts for efficacy in clinical trials. Nevertheless, these challenges are known and can be addressed in order to internationally pool existing data and to give patients the opportunity to contribute own data to achieve the overall goal of trial readiness in a rare disease. For a majority of parents and patients, the knowledge of helping to advance new therapies for future effected families by providing own data on the course of disease, helps in coping with these devastating progressive diseases like NCL. The motivation of these families is therefore high in order to help for a greater good and should be addressed by making it possible that not only patients in excellent health care systems are able to join via expert centers, but are able to provide needed data by themselves that can be validated centrally by experts in the field.

International data collection collaborations had been slowed across all diseases by the new European Data Protection Regulations (GDPR) that came into effect in May 2018. The development of new guidelines on sharing data had to be implemented, local ethic approvals renewed, and individual patients had to be re-consented for international collaboration on sharing of data. Subsequently this may have helped to streamline the collaborative use of identical tools for evaluating the course of disease and the type of data collected. In using the same tools, these can be developed further and adapted to individual center needs, moreover though, it makes comparison between centers objective and evaluation of data sets uncomplicated.

In order to be able to use data acquired in clinical settings for use in clinical trials, these need to meet the same quality requirements as data collected in clinical trials (monitorable) in order to be auditable and approved by health care authorities. Written reports signed with date and digital records that have audit trails implemented within the systems are standards that need to be in place.

Evaluating rare diseases and quantifying progression of disease nevertheless remains challenging and has its limitations in use. One challenge is the limited sample size in such diseases, another the mostly retrospective nature of longitudinal data with varying timepoints of data collection and its retrieval from medical charts and country dependent obligatory childhood exams.

Nevertheless, these challenges can be overcome. The natural history data collection in late-infantile CLN2 disease by Nickel et al. (42), investigated the largest cohort so far for late-infantile CLN2 disease of quantitative natural-history data, acquired in a multi-site international setting. Before this collaboration, data describing the natural history of late-infantile CLN2 disease were very limited: previous studies either contained mostly retrospectively collected data or were restricted to cross-sectional data only. Moreover, these previous studies analyzed data sets from one center only without providing evidence that the data were representative across multiple study sites and in an international setting. Thus, the data from this natural history study represent an important milestone in quantifying the progression of neurodegeneration in children with late-infantile CLN2 disease according to an editorial comment by Mink in 2018 (44).

Data from this study have successfully served as natural-history control data in completed experimental therapy trials on intraventricular ERT for CLN2 disease (43) and are also being used in ongoing experimental therapy trials on intraventricular ERT (NCT01907087, NCT02485899, NCT02678689) and intraparenchymal adeno-associated gene therapies [(45), NCT00151216, NCT01161576, NCT01414985]. Intraventricular ERT has recently been approved in the US, Europe and Japan as treatment for CLN2 disease [(46–48), online] on the basis of comparisons with these natural-history data. They will also be valuable for any clinical trials looking at future treatment developments for CLN2 disease such as other forms of gene therapy or new pharmacological approaches.

## Author Contributions

MN and AS contributed to conception and design of the study, writing of the manuscript as well as manuscript revision, read, and approved the submitted version.

## Conflict of Interest

The authors declare that the research was conducted in the absence of any commercial or financial relationships that could be construed as a potential conflict of interest.

## Publisher's Note

All claims expressed in this article are solely those of the authors and do not necessarily represent those of their affiliated organizations, or those of the publisher, the editors and the reviewers. Any product that may be evaluated in this article, or claim that may be made by its manufacturer, is not guaranteed or endorsed by the publisher.
